# How museum-based creative-product experiences shape cultural confidence via cultural cognition and cultural identity

**DOI:** 10.3389/fpsyg.2025.1742322

**Published:** 2026-01-15

**Authors:** Jinjun Xia, Yashi Li, Yuanheng He, Ke Ma

**Affiliations:** 1 Department of Design, School of Arts, Chongqing University, Chongqing, China; 2 Industrial Design Center, Chongqing University, Chongqing, China; 3 Faculty of Social Sciences, University of Macau, Taipa, Macao SAR, China

**Keywords:** cultural and creative tourism product design, cultural cognition, cultural confidence, cultural identity, museum context, people–place bonds, place-based experiences

## Abstract

**Introduction:**

Cultural confidence plays an important role in cultural development, social harmony, and personal growth. As living standards improve, demand for cultural and creative tourism products continues to rise because they provide meaningful, place-based experiences in built cultural environments such as museums. Framed within scholarship on people–place bonds, this study examines how cultural and creative tourism product design (CCTPD)—as a cultural communication carrier situated in a museum context—is associated with cultural confidence, and whether these associations are consistent with a sequential pattern involving cultural cognition and cultural identity.

**Methods:**

Using 314 survey responses collected in the Cultural and Creative Experience Pavilion of the Chongqing Three Gorges Museum, structural equation modeling (SEM) tested direct and indirect associations among cultural cognition, cultural identity, and cultural confidence. Given the cross-sectional design, the estimated mediation paths are interpreted as correlational rather than causal. Drawing on the Strategic Experiential Modules (sense, act, think, relate, feel), CCTPD was modeled as a higher-order construct with five first-order dimensions.

**Results:**

SEM results were consistent with a sequential pattern in which cultural cognition was positively associated with cultural identity, which in turn was positively associated with cultural confidence. CCTPD was positively associated with cultural confidence through multiple direct and indirect pathways, with cultural identity serving as a key mediator. Dimension-level analyses indicated that sense, act, and think experiences were associated with cultural cognition; think and relate experiences were associated with cultural identity; and sense, relate, and feel experiences were associated with cultural confidence.

**Discussion:**

Situated within a museum as a salient cultural place, the findings suggest that designed place-based experiences may relate to cultural confidence primarily through identity-related processes. Practically, designers and curators can leverage sense/act/think features to suggest cognition, emphasize think/relate to strengthen identity, and activate sense/relate/feel to support confidence—informing environmental psychology, design strategy, and policy/planning for places seeking to nurture identity processes under social and cultural change.

## 1 Introduction

In the context of globalization, cultural confidence is an important means of preserving national cultural characteristics and promoting cultural innovation. Cultural confidence refers to a collective cultural identity, a sense of belonging, and affection, grounded in an individual’s profound understanding, acceptance, and practice of their own culture. Such confidence serves as a powerful spiritual driver, promoting specific value orientations and positive behaviors (Wan and Rucker, 2013). From a social perspective, cultural confidence, as a phenomenon in social psychology, is critical with respect to efforts to promote cultural development and social harmony (Zhou and Bi, 2020). On an individual level, cultural confidence can shape an individual’s worldview, thereby playing a crucial role in maintaining mental well-being (Douglas et al., 2008).

The generation of cultural confidence involves psychological processes such as cognition, emotion, and attitude. Theoretically, many scholars have analyzed the psychological characteristics of cultural confidence. Wang and Huang (2018) suggested that the development of cultural confidence follows three psychological stages: cognitive, emotional, and volitional processes. Considering the characteristics of a fluid society, Chen and Wei (2020) proposed a three-dimensional psychological structure model of cultural confidence, encompassing cognition, emotion, and motivation. Empirically, existing cultural confidence scales focus on two dimensions: pride and admiration for culture (Zhou and Bi, 2020) and individual self-efficacy (Pan et al., 2021). Once abstract variables like cultural confidence are quantified, scholars have applied structural equation modeling to explore causal relationships among variables, enabling the transformation of dimensions into mechanisms. In different fields, cultural confidence has been studied as either an outcome variable or a mediating variable to examine its impact, such as its role in promoting citizen behavior (Yin et al., 2023) and residents’ well-being (Pan et al., 2021) or cultural loyalty (Lin et al., 2022).

Overall, these studies have highlighted the dynamic nature of the psychological development of cultural confidence and emphasized the crucial roles of cultural cognition and cultural identity in shaping its psychological foundation. This research has laid a theoretical and methodological foundation for exploring the psychological mechanism underlying the generation of cultural confidence. However, as a psychological characteristic, cultural confidence has only recently emerged as a popular topic (Li and Kim, 2023). Some previous research on this topic has focused on broad theoretical investigations across various social, political, and educational fields (Wang and Huang, 2018; Guan, 2023), by considering the definition, dimensions, and social significance of cultural confidence. Other studies have treated cultural confidence as a variable in quantitative analyses. When the abstract concept of cultural confidence is quantified (Zhou and Bi, 2020; Pan et al., 2021), scholars in different fields take cultural confidence as an outcome variable or an intermediary variable, explore the causal relationship between cultural confidence and other variables using structural equation modelling (SEM), and consider the transformation from dimensions to mechanisms (Lin et al., 2022; Zhou et al., 2022). However, few studies have tested a theoretically grounded psychological pathway to cultural confidence within a specific situational context.

To foster cultural confidence, it is essential to have access to opportunities and channels for learning about outstanding traditional culture. Cultural and creative tourism products, developed through creative design based on the cultural and tourism resources of specific destinations, represent a combination of market-oriented products and services designed to meet users’ emotional needs (Gao and Xu, 2022). As a significant derivative of cultural tourism, these products are not only an indispensable component of cultural tourism but also serve as a key tangible medium for individuals to engage in cognition and reflection, thereby stimulating the psychological development of cultural confidence. However, previous research on cultural and creative tourism products mainly focused on the consumer perspective (Bynum Boley et al., 2013) and design methods (Song et al., 2021). Specifically, this mainly focused on the relationships among cultural connotations (Feng et al., 2023), emotional design (Yu et al., 2022), experience design (Yen, 2022), and the psychological dimensions of cultural confidence. Although prior research highlights the importance of cultural and creative tourism product design (CCTPD) for cultural confidence and the need to probe culture’s intrinsic and metaphysical dimensions while advancing innovation (Gao and Xu, 2022; Chen and Lei, 2023), the interrelations and operative mechanisms among these factors remain unclear. Such studies have focused mainly on the single psychological dimension of emotional identity or on certain specific elements of creative design.

Therefore, this study adopted an empirical research paradigm to investigate the psychological mechanisms that emerge following users’ experiences with cultural and creative tourism products. This study thus investigated the mechanisms underlying the psychological generation of cultural confidence and the impacts of different design dimensions and specific design elements of cultural and creative tourism products at various stages of their formation. First, this study combined theories of cultural psychology, summarized relevant concepts and the different connotations of confidence in the literature, and identified the psychological mechanism underlying the generation of cultural confidence. Second, relevant theories and methods of design were used to identify existing design elements of cultural and creative tourism products, and were classified into five dimensions (i.e., SENSE, FEEL, ACT, THINK, and RELATE) based on SEMs, forming a dimensional model of the design elements of the cultural and creative tourism product experience. This study further developed a theoretical model of the psychological mechanism underlying cultural confidence and the design experience dimension of cultural and creative tourism products and proposed a path hypothesis. Finally, this study collected a large amount of questionnaire data through empirical research, adopted SEM to verify the hypothesis, and presented limitations and suggestions for future research. Specifically, this study explored the following questions:

- What is the psychological generation mechanism of cultural confidence? How are cultural cognition, cultural identity, and cultural confidence related in this process?
- How does CCTPD influence cultural cognition and cultural identity, thereby affecting cultural confidence?
- Specifically, how do different design dimensions of CCTPD influence the psychological generation of cultural confidence through cultural cognition and cultural identity?

Through this multidisciplinary theoretical approach, this study aims to provide empirical evidence supporting the interaction between design and psychology, serving as a valuable reference for other scholars exploring similar topics. Furthermore, by examining how CCTPD shapes the development of cultural confidence, this study offers practical guidance for designers in creating culturally influential products, thereby increasing product value. Additionally, the study provides practical guidelines and design strategies for both designers and policymakers. These guidelines aim to promote cultural dissemination and effectively enhance consumer cultural confidence. Accordingly, the study seeks to promote the sustainable development of the cultural industry and protect cultural diversity.

## 2 Literature review

### 2.1 The psychological generation of cultural confidence

Confidence is a complex psychological construct that can be conceptualized as hierarchically structured. It reflects individuals’ self-affirmation and a relatively stable disposition to appraise their abilities and value in a favorable way (Chai et al., 2024). Confidence reflects a person’s perceived capacity to manage life demands and pursue desired outcomes, offering reassurance about achieving both psychological and material goals (Kamas and Preston, 2012). In the psychological context, culture is defined as a network of shared knowledge that is produced, disseminated, and reproduced among a group of interconnected individuals. The core function of culture is to offer a common reference framework that enables individuals to understand social realities, engage in social interactions, and adapt to external environments (MacWhite, 1954). Therefore, strengthening attachment to one’s cultural roots may reinforce trust in that culture and, in turn, support cultural confidence (Pan et al., 2021).

Structurally, cultural confidence is homologous to other self-related concepts that include similar psychological components. The study of cultural confidence requires a thorough understanding of the concept of confidence. Therefore, this study aims to clarify the fundamental connotations and basic attitude of cultural confidence by identifying similar concepts and different connotations of confidence. This study examines the generation of confidence by referring to different fields to understand the background of cultural confidence.

#### 2.1.1 The relationships among concepts similar to confidence and cultural confidence

Before exploring cultural confidence, it is necessary to identify several key concepts related to confidence and their interrelationships. These concepts are similar to confidence but focus on different aspects, offering a more comprehensive understanding of the multidimensional nature of cultural confidence and clarifying its essence.

1. Self-esteem and confidence: Self-esteem generally refers to an individual’s overall evaluation of their worth and abilities. It pertains to an individual’s intrinsic values and self-acceptance and reflects the degree to which an individual likes or dislikes themselves (Dipboye, 1990). Unlike confidence, self-esteem is not related to past performances or experiences. Confidence, on the other hand, is more closely related to the individual’s trust in their specific abilities or the outcomes of their future actions. Self-esteem is a broader form of self-assessment, whereas confidence is more specific. Accordingly, cultural confidence is established through the cultural subject’s understanding and reflection on their own culture. This process involves cognitive elements and critical reflection rather than merely reflecting the individual’s attitudes towards the value and vitality of their own culture.
2. Self-efficacy and confidence: Self-efficacy refers to an individual’s belief in his or her ability to complete specific tasks (Bandura, 1977). It focuses more on the individual’s belief in their capabilities in particular areas or activities, such as swimming or weightlifting, which can be observed and perceived (Gist and Mitchell, 1992). Confidence is a broader concept that encompasses not only the evaluation of abilities but also the individual’s degree of confidence in the outcomes generated by those abilities, as well as the strength of the belief. Accordingly, cultural confidence includes not only the cultural subject’s affirmation of the value and vitality of their own culture but also their belief in the transmission and continuity of culture. This notion should not be limited to an individual’s belief in their ability to engage in cultural activities.
3. Trust and confidence: Trust usually refers to reliance and dependence on others or systems. It involves making judgments about the similarity between oneself and others or systems in terms of intentions or values, as well as about the individual’s expectations of beneficial outcomes based on perceived similarities among individuals (Earle, 2009). However, confidence is more closely related to individuals’ beliefs in their abilities and the outcomes of their future actions; it thus indicates a greater reliance. Therefore, cultural confidence refers to the cultural subject’s evaluation of and belief in the value and vitality of their culture, extending beyond mere perception of consensus.

In summary, the connotations of confidence in the context of cultural confidence differ from those of other related concepts in three main ways: confidence involves a reflective process, thus distinguishing it from self-esteem; it includes affirmative attitudes and beliefs in the overall value and vitality of one’s culture, thus differentiating it from self-efficacy; and it encompasses higher levels of reliance and pride, thus differentiating it from trust.

#### 2.1.2 The relationships among different types of confidence and cultural confidence

These definitions, which range from a focus on cognitive judgement to an emphasis on emotional states, provide different perspectives on the essence of confidence. This helps us to clarify the orientation of cultural assertiveness and the factors that may stimulate its production.

(1) Confidence as judgement: Confidence is regarded as pertaining to personal judgement regarding future events or outcomes, thus emphasizing its association with decision-making processes (Bertana et al., 2021).
(2) Confidence as conviction/certainty: Confidence is viewed as related to the individual’s conviction or degree of certainty in the beliefs or attitudes they adopt and thus as involving the individual’s subjective awareness of their own state (Oney and Oksuzoglu-Guven, 2015).
(3) Confidence as assessment: Confidence refers to the process of evaluating past and present evidence based on existing clues and meta-cognitive beliefs to predict future outcomes. In psychological experiments, a person’s confidence is often assessed by eliciting confidence judgments (Double and Birney, 2024).
(4) Confidence as belief: Confidence is viewed as a belief that is based on experience or evidence (such as past experiences), including the belief that specific future events will occur in the expected manner (Oney and Oksuzoglu-Guven, 2015).
(5) Confidence as a feeling, perspective, and expression: According to this definition, confidence involves a feeling of self-assurance and a lack of anxiety. It can also be regarded as a favorable judgment associated with subjective probability, often described as a feeling of certain expectation or an open expression of perceived likelihood, as well as a favorable perspective on subjective probabilities or an open expression of possibilities (Barbalet, 1996).

In summary, the definition of cultural confidence closely aligns with the concept of “confidence as belief.” Cultural confidence involves deep-seated beliefs regarding and identification with a culture based on an understanding and evaluation of that culture’s history, values, achievements, and characteristics. Correspondingly, the belief in cultural confidence is grounded in cultural experiences and historical evidence that shape the unique value and significance of a specific culture. Moreover, cultural confidence extends beyond individual confidence to encompass the collective confidence of a societal group in its cultural traditions and future, reflecting positive evaluations and expectations for the group’s cultural identity and continuity. This study proposed that, at the psychological level, cultural confidence arises from an individual’s profound understanding of cultural value, uniqueness, and vitality, coupled with a positive identification with it. It encompasses not only confidence and admiration for culture but also self-confidence, accompanied by an internalized belief system and externalized practice. It is characterized by four key aspects: cognitive understanding and reflection, a sense of pride, a sense of conviction, and internalized practice.

#### 2.1.3 Derivation of the psychological mechanism underlying cultural confidence

The concept of confidence in cultural confidence is both distinct from and closely related to other confidence-related concepts. Drawing on commonalities in social psychology, research in these areas offers valuable insights into the psychological structure and mechanisms underlying the generation of cultural confidence.

In the domain of sports, Vealey et al. (1998) argued that sports confidence differs from general confidence. Sports confidence results from the interactions between specific sports and athletes’ personality traits. Organizational culture and athletes’ characteristics influence the sources of athletes’ confidence, which in turn affect their levels of sports confidence and performance outcomes. Furthermore, the performance outcomes influenced by sports confidence provide feedback that shapes the sources of athletes’ confidence, thus creating a closed circle of sports confidence extending from generation to effect and back to reproduction. In the field of rehabilitation, Dennis (1999) highlighted the importance of developing breastfeeding confidence grounded in self-efficacy. According to social learning theory, postpartum women enter psychological states that reflect efficacy information obtained through psychological reflection; in turn, these states influence their breastfeeding behavior.

The generation of confidence across the fields explored in the literature thus reveals a common characteristic: confidence is generated through a cognitive process followed by reflective processing. The receipt of external information constitutes the cognitive process, and the development of a positive attitude towards a specific task or situation through reflection constitutes the emotional process. This emotional process is further influenced by both internal and external factors and results in confidence in one’s ability to perform a specific task. Internal factors primarily refer to the distinct characteristics of individuals within a specific domain, which can lead to different outcomes at different stages of confidence formation. On the other hand, external factors are uncontrollable variables, such as cultural context or the influence of other parties, that can affect the individual’s cognitive and reflective processes.

Therefore, this study concludes that the core components of the mechanism underlying confidence generation can be characterized as a process that leads from cognition to emotion and ultimately to confidence; both external factors and internal reflection influence this process. Different fields might be affected by other variables or specific factors. Regarding the definition and content of cultural confidence, the mechanism underlying its generation can be viewed as leading from cultural cognition to cultural identity and ultimately to cultural confidence. This process is influenced by individuals’ internal characteristics and external cultural factors; in this context, this study considers CCTPD as an external cultural stimulus.

### 2.2 Five dimensions of CCTPD based on SEMs

Due to the converging influences of cultural and tourism integration, digital empowerment, and the experience economy, the demand for cultural and creative tourism products is shifting from a focus on “functional use” to an emphasis on the “cognitive experience of information” (Song et al., 2021). Consumers are no longer purchasing mere commodities but rather a cultural experience or a form of spiritual satisfaction that provides them with a better product (Su et al., 2020). Therefore, it is necessary to shift away from the standardization of tourism products to propose a new model for current cultural and creative tourism products, namely, a model that includes both tangible content and intangible services, thereby expanding the scope from physical products to service experiences that are not only unique but also multidimensional (Chen et al., 2022).

Experience marketing was first proposed by Schmitt. He divided experiences into sense, feel, think, act, and relate, thereby developing the notion of strategic experience modules (SEMs), which focus on the consumer (Schmitt, 1999). Based on the SEMs model, the five dimensions of CCTPD are defined as follows: (1) Sense experience stimulates tourists’ sensory perceptions through design elements involving vision, smell, hearing, taste, and touch, thereby satisfying sensory needs; (2) Think experience integrates knowledge and informational content to support cognitive engagement during use, meeting learning-related demands; (3) Act experience focuses on functional features aligned with consumers’ behavioral patterns, supporting lifestyle integration; (4) Relate experience promotes social interaction and sharing through design, often leveraging social platforms to facilitate resonance, meeting social sharing needs; and (5) Feel experience evokes emotional resonance by connecting tourism experiences with local culture, addressing affective needs.

Recent studies highlight that immersive technologies (e.g., VR/AR and interactive installations) are associated with richer museum experience trajectories, for example by supporting perceived enjoyment, concentration, and perceived usefulness, which are in turn related to visitors’ attitudes and behavioral intentions. For example, Liu and Sutunyarak (2024) integrate TAM and flow theory to explain how immersive technology relates to visitors’ satisfaction and behavioral intention, underscoring the relevance of technology-enabled sense/feel/think/relate attributes in museum contexts.

To situate this study within the broader visitor-studies and museum-experience literature, we also draw on relevant international perspectives. Beyond the Chinese context, visitor studies and museum experience research have increasingly emphasized how learning, communication, and engagement emerge from the interaction between personal, sociocultural, and physical/technological contexts (e.g., Davidson, 2025). Recent empirical work also examines how visitors’ experiences inform museums’ communication strategies and service design (Li, 2024), and how the physical context shapes attention and engagement during museum learning (Rainoldi et al., 2020). Incorporating these perspectives helps position the present model within broader international debates on museum-based experiences and identity-related outcomes. These international insights also align with our SEM-based framing of CCTPD as multidimensional experience design, which we further test in the proposed cognition–identity–confidence model.

## 3 Hypotheses development

### 3.1 Psychological generation of cultural confidence

Psychological development progresses gradually from lower to higher stages. Cultural cognition is the first step in forming cultural identity and cultural confidence. Only by actively understanding a cultural object and developing a certain level of cultural self-awareness can one form the basis for positive attitudes and confidence toward that culture. Cultural cognition refers to the views and attitudes individuals develop after processing cultural information, reflecting how they understand a given culture (Wang and Huang, 2018). Building on Guan (2023), cultural cognition can be viewed as shaped by both internal and external components. Internally, the “mind” provides the core cognitive basis, while factors such as cultural background and emotions can influence how cultural information is processed and evaluated, highlighting individual subjectivity and agency. Externally, the cultural environment provides contextual cues that shape how cultural symbols are interpreted and how cultural meanings are constructed. Therefore, cultural cognition not only depends on the mind but is also influenced by social and cultural factors, making it a subjective process that carries specific social attitudes.

From a psychological perspective, attitude is an evaluative response tendency formed by an individual based on certain beliefs (Wang and Huang, 2018). Cultural identity, as an attitude, manifests as an individual’s or group’s sense of belonging and emotional identification with a specific culture. According to social identity theory, individuals define themselves through group membership and, under the influence of social and cultural factors, form a unified cognition of the world (Newman et al., 2007). Cultural cognition is the process by which individuals understand cultural symbols and meanings. This process interacts with the cultural environment, laying the foundation for the formation of cultural belonging and identity. Through emotional identification with culture, individuals strengthen their identification and pride in their own culture, further enhancing cultural identity. Wan and Chew (2013) suggested that acquiring cultural knowledge enables individuals to construct a cultural identity. Li and Liu (2022) reported that users perceive various sub-dimensions of destination culture, such as institutional, spiritual, and material culture, and spontaneously generate a cultural identity. Similarly, Zong et al. (2023) found that the stronger an individual’s understanding of traditional cultural symbols, the greater their cultural identity and emotional connection to those symbols. Hence, this study put forth the following hypothesis:

*H1:* Cultural cognition significantly affects cultural identity.

Cultural confidence is a process of simultaneous “knowing and doing,” in which “knowing” provides the content foundation for psychological development, and “doing” represents the externalization of this confidence (Guan, 2023). In the relationship between cultural cognition and cultural confidence, individuals who gain a deep understanding of cultural history and spiritual values can recognize the uniqueness of their culture, which leads to a strong sense of belonging and pride, thereby laying the foundation for greater cultural confidence. Moreover, cultural confidence is a positive emotional expression of an individual’s psyche. According to Ramalho and Forte (2019), knowledge and cognition significantly enhance an individual’s self-confidence and self-efficacy. Therefore, through cultural cognition, individuals strengthen their sense of control and identification with their culture, which not only boosts psychological confidence but also promotes the practical application of cultural elements. Positive feedback from these practices further reinforces cultural confidence. Ghaderi et al. (2023) suggested that cultural confidence is based on a comprehensive understanding and knowledge of a given culture. Similarly, Zhou et al. (2022) reported that understanding the history of the Chinese nation, particularly the corresponding tourists who followed, and the pride in being Chinese, helped to enhance pride in Chinese culture. Hence, this study put forth the following hypothesis:

*H2:* Cultural cognition significantly affects cultural confidence.

Identity refers to people’s recognition of certain things, roles, or cultures (Lin et al., 2022). It is a psychological process that moves from cognition to emotion and then influences individual behavior. Cultural identity, which is equivalent to social identity in a cultural context, is part of an individual’s self-definition (Chattaraman et al., 2009) and highlights the psychological connection between an individual and a culture (Wan and Chew, 2013). Regarding the structure and measurement of cultural identity, Keillor et al. (1996) identified four dimensions of ethnic identity: cultural homogeneity, belief structure, ethnic heritage, and ethnocentrism. Wang and Hu (2011) argued that cultural identity encompasses symbols, identity, and values. Therefore, existing measures of cultural identity focus primarily on its materiality, group affiliation, and emotional aspects. Materiality refers to people’s tendency to recognize both material and immaterial elements of cultural carriers, emphasizing the content of cultural identity; group affiliation emphasizes individuals’ evaluation and sense of belonging to cultural groups in social relationships, reflected in behaviors and convergence with the target group; emotionality refers to the degree to which individuals accept social norms and values, reflecting self-recognition and pride in their own national culture.

From an individual perspective, cultural identity is developed by understanding and engaging with cultural values, thereby reflecting a positive stance toward and affirmation of those values (He and Wang, 2015). Individuals who are attracted by a cultural phenomenon or piece of cultural content are very willing to share their feelings and insights with others (Wang and Huang, 2018). When a person identifies strongly with a culture, they are inevitably loyal to it and integrate its core into their psychological and spiritual being; this process embodies the essence of cultural confidence (Lin et al., 2022). Furthermore, extensive research has suggested that cultural identity is a prerequisite for cultural confidence (Black, 1998; Lin et al., 2022), and as the consumers’ sense of cultural identity increases, their cultural confidence is also enhanced (Cleveland et al., 2015; Sun, 2022; Zhou et al., 2022). Hence, this study put forth the following hypothesis:

*H3:* Cultural identity significantly affects cultural confidence.

### 3.2 The relationship between CCTPD and cultural cognition, cultural identity, and cultural confidence

In the context of tourism, tourists can gain a comprehensive understanding of a given cultural entity by purchasing cultural products or visiting a digital cultural experience museum. By engaging in cultural consumption, users can experience another culture and create cultural memories that construct and reshape local cultural identity (Chen et al., 2023). Culture influences perception, which in turn affects cognition at both the social and individual levels. Perception and cognition depend on sensory inputs at the individual level and involve various top-down processes (Kastanakis and Voyer, 2014). Cultural and creative tourism products are tangible carriers that can enable individuals to perceive and experience culture, thereby stimulating the psychological process associated with cultural confidence. According to the theory of hierarchical needs, the level of consumer experience is closely related to the level of individuals’ needs (Yang, 2022); thus, the process by which users obtain in-depth experience with products can lead to the emergence of different psychological stages.

Cultural cognition involves tourists’ understanding and mastery of cultural knowledge. According to information processing theory, the cognitive process includes stages such as information reception, selective attention, encoding, storage, and retrieval (Payne, 1980). In the design of cultural and creative tourism products, design elements (such as graphics, symbols, text, and colors) are perceived by tourists as external information. These designs help focus tourists’ attention through multi-sensory stimulation, enhancing the understanding and memorability of the information. For example, Song et al. (2021) found that introducing cultural symbols, interactive displays, and contextual designs can attract tourists’ attention, thereby facilitating information encoding and storage.

Furthermore, as discussed in earlier analysis of the cultural cognition system, individuals’ background knowledge and past experiences influence how they process external information. In the context of cultural and creative tourism products, tourists may combine their own cultural background and travel experience to interpret these cultural elements. This process also contributes to their cultural cognition.

Cultural identity emerges when individuals connect with a culture, especially when their personal values align with it, leading to a strong sense of belonging. According to social identity theory, self-identity is not only shaped by personal traits but also by the groups and cultural contexts to which individuals belong (Spears, 2011). Cultural and creative tourism products convey core cultural values through design elements such as totems, cultural stories, and historical symbols. These elements serve as vehicles for cultural knowledge and help tourists connect emotionally with the culture, fostering a sense of cultural identity. For instance, Feng et al. (2023) demonstrated the historical continuity of local culture by incorporating redesigned elements of Wuqiang’s new year paintings into clothing design. The emotional value embedded in these designs, such as the historical memory of Wuqiang’s new year paintings, resonates with the local community. In conclusion, cultural and creative tourism products promote cultural identity through cognitive understanding, group belonging, and emotional connection.

Cultural confidence is not only a continuation of cultural cognition and identity, but also a deep psychological identification and emotional pride. Cultural and creative tourism products, through their design and experience, not only help visitors understand and identify with a particular culture but also inspire their cultural confidence. This is especially true when cultural carriers with spiritual or ethnic identity are selected. These products can evoke emotional resonance, stimulating visitors’ cultural identity and pride, thereby fostering a sense of “cultural mission” or “cultural honor.” For example, Zhou et al. (2022) found that red tourism, by immersing visitors in environments with revolutionary historical backgrounds, enhances their emotional connection to local culture and cultural confidence.

Additionally, another source of cultural confidence comes from the mechanism of cultural comparison. According to social comparison theory, individuals evaluate and understand the value of their own culture by comparing it with other cultures (Koenig-Lewis et al., 2021). Cultural and creative tourism products, especially in cross-cultural or multicultural contexts, encourage visitors to discover and identify with the advantages of their own culture, thus enhancing their cultural confidence.

Lin and Wang (2022) found that experiencing cultural products promotes cultural cognition and emotional identification among experiencers, as evidenced in their study on the experiential value participants perceived in the intangible heritage of Cantonese embroidery. Koenig-Lewis et al. (2021) reported that travelers can gain knowledge and enhance their cultural identity by experiencing local festivals. Similarly, based on David’s “cultural experiential learning circle,” Yan and Dong (2023) explored the pathways to the generation of cultural identity. Based on an exploration of the relationship between cultural experience and cultural confidence, Chen (2020) reported that college students’ experiential encounters in the context of creative design enriched their understanding of culture and enhanced their cultural confidence. Gao and Xu (2022) clarified the important roles of experiential design in cultural and creative tourism products in cultural cognition, cultural confidence, cultural dissemination, and cultural development, based on a case analysis of the Fuchun Mountains E-post. Based on the above analysis, this study proposes the following hypotheses:

*H4:* CCTPD positively affects cultural cognition.

*H5:* CCTPD positively affects cultural identity.

*H6:* CCTPD positively affects cultural confidence.

**Figure 1:**
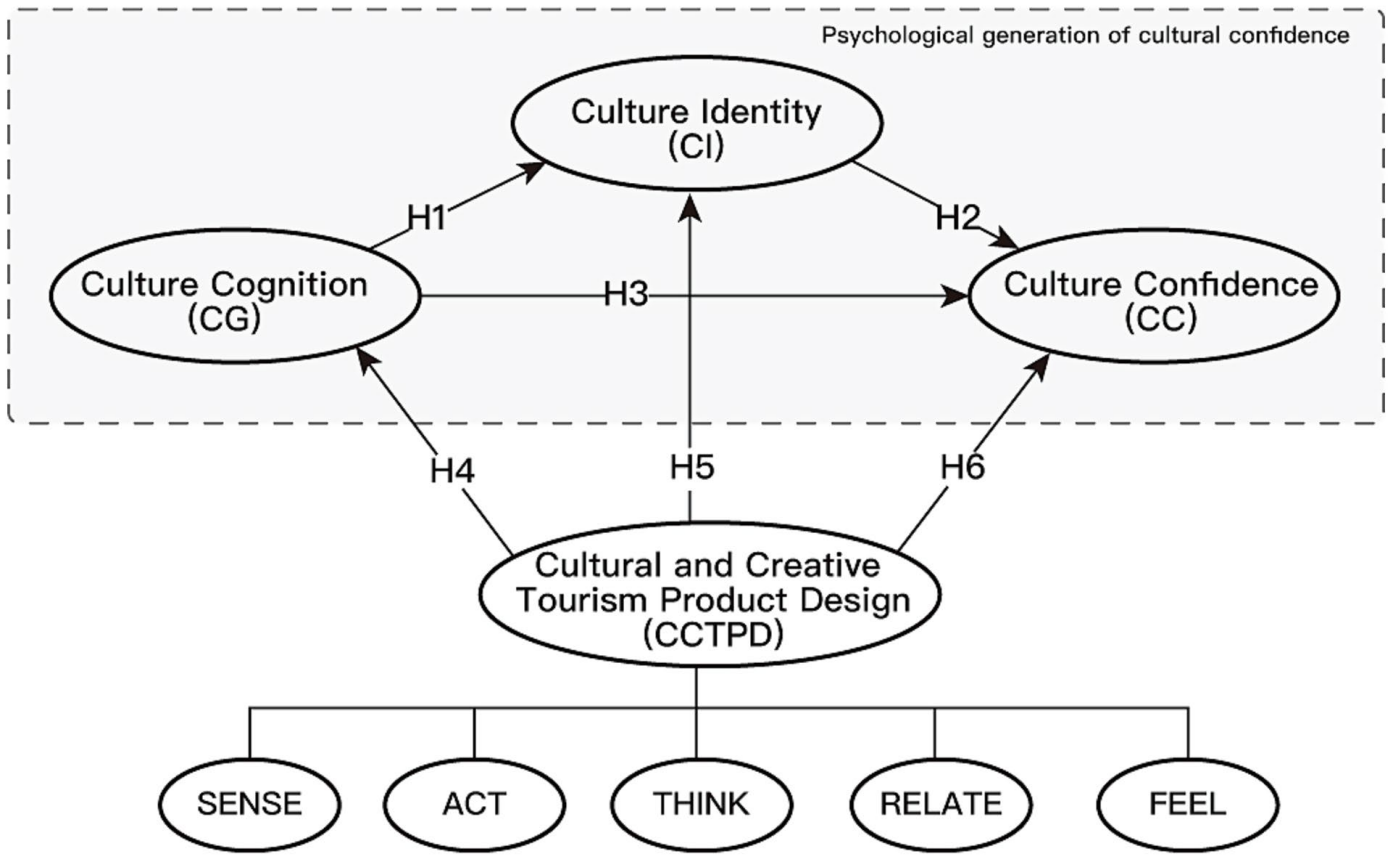
Proposed conceptual model.

Based on the research findings discussed above and the comprehensive reasoning underlying this study, the psychological generation of cultural confidence may involve a process of moving from cultural cognition to cultural identity, or it may be directly stimulated by cultural cognition. CCTPD, composed of sense, act, think, relate, and feel experience, may directly affect the different stages of the psychological generation of cultural confidence. Therefore, the following model is constructed (see Figure 1).

## 4 Data and methodologies employed

### 4.1 Study site

This study was conducted at the Cultural and Creative Experience Pavilion of the Chongqing Three Gorges Museum. The Chongqing Three Gorges Museum, one of China’s first-level national museums, is an important cultural landmark in Chongqing. It showcases Three Gorges culture, Ba culture, and local Chongqing culture, attracting numerous domestic and international visitors. Unlike traditional museums, the Three Gorges Museum emphasizes integrating modern technology and interactive experiences. It utilizes digital displays and multimedia technologies to enhance visitors’ sense of immersion. As an innovative expansion, the Cultural and Creative Experience Pavilion not only displays traditional historical artifacts but also offers a variety of cultural and creative tourism products and interactive experiences to help visitors deeply appreciate the unique charm of Three Gorges culture. The Experience Pavilion features 377 types and 461 styles of cultural and creative tourism products, employing the latest digital interactive technologies to provide visitors with an immersive experience of local culture, fostering emotional resonance and cultural identity. The Pavilion not only offers rich cultural content but also enhances visitors’ sense of participation and experience through its fun, innovative designs. The annual sales of cultural and creative tourism products reach approximately 8 million yuan, and visitors’ consumption behavior of tourism-related creative products reflects their recognition and confidence in local culture. Additionally, a large body of academic research on cultural and creative tourism products and design has been conducted based on the Three Gorges Museum, making it a highly representative and feasible research site.

### 4.2 Instruments

#### 4.2.1 Construction of the evaluation index of CCTPD

The function of design elements is to carry and transmit culture. These elements thus serve as important evaluation criteria for whether cultural and creative tourism products meet users’ experience expectations (Jin et al., 2015). In recent years, scholars have proposed various design elements and evaluation criteria for cultural and creative tourism products from various perspectives.

Regarding the impact of users’ cognitive arousal and consumption preferences, Song et al. (2021) proposed design principles rooted in the thematic, participatory, and diverse nature of experiential cultural tourism product design models grounded in cognitive psychology. Tu et al. (2019) summarized 15 attributes of creative cultural products and used the cultural elements of the Forbidden City as an example in an exploration of consumers’ cultural cognition and preferences. Chai et al. (2015) adopted cultural hierarchy theory. They reported that consumers prefer cultural products with “inner” and “behavioral” metaphysical connotations over “outer,” “tangible” elements, such as typical local cultural elements and emotionally resonant cultural elements. Similarly, Yang et al. (2022) referenced cultural level theory and reported that design students are more likely to perceive culture at the material level.

In terms of emotional identification, given the multidimensional nature of emotions, Yu et al. (2022) identified key factors influencing emotional identification with cultural and creative tourism products, including meaningfulness, pleasure, practicality, reliability, usability, and convenience. Feng et al. (2023) suggested that localization, uniqueness, and different designs in terms of shape, color, and volume, especially with respect to beauty and elegance, are important factors in expressing geographical cultural identity. Yen (2022) emphasized the importance of cultural emotion and interaction in the context of a sustainable festive event. Experience is closely linked to users’ cultural psychology, and cultural and creative design elements serve as carriers of user experience, stimulating different psychological stages of cultural understanding through experiential forms. In addition, Chen and Lei (2023) found that a focus on emotional experiences characterized by uniqueness, resonance, aesthetics, and fun could align with users’ cultural cognition and identification motives.

Therefore, various design indicator elements associated with the cultural and creative tourism products explored in this study are proposed based on a mapping of the relationships between the design elements of these products and experiential design. This approach can help reveal the potential needs of tourism consumers and apply them to the experiential design of cultural and creative tourism products. This paper lists and analyses the design elements associated with cultural and creative tourism products that different scholars have proposed and classifies them into sense experience, act experience, think experience, relate experience, and feel experience in line with the SEMs proposed by Schmitt (see Table 1), thereby developing preliminary evaluation indicators for CCTPD from an experiential perspective (see Table 2).

**Table 1:** Classification of design elements based on the SEMs.

Source	Dimensions of the SEMs				
	SENSE	ACT	RELATE	THINK	FEEL
Song et al. (2021)	Uniqueness, diversification, novelty	Participation	Service	Theme	–
Yang et al. (2022)	Color, dermatoglyphic pattern, material, form, structure, craft, historical features, geographical features	Operation, function	–	Legend and allusion, custom, religion, and philosophical thinking	Spiritual values
Feng et al. (2023)	Uniqueness, originality, beauty, elegance	–	–	Localization, culture/civilization	Friendliness
Yu et al. (2022)	–	Practicality, reliability, usability, convenience	–	–	Meaningful, pleasurable
Tu et al. (2019)	Cultural traits, local characteristics, aesthetic, and unique creativity		Cultural sustainability	Cultural symbol, traditional culture, historical memory, cultural inheritance,	Cultural identity, sensual interest, fashion trend, cultural connotation
Chen and Lei (2023)	Uniqueness, beauty	Interactivity, practicality, durability, usability	Sociability, cost-effectiveness, shareability	Cultural memory, collection-related value, representation, cultural popularization, educational value	Emotional healing, emotional resonance, fun, fashion trend
Chai et al. (2015)	Colors, profiles, texture, craftsmanship, method of presenting, typical	Better designed perception, using patterns, functionality	–	Story-like, cultural meaning, appeal	Familiarity, emotional resonance
Yen (2022)	Aesthetic, place identity	Safety	Centrality, self-expression, love, and belonging	Educational, attraction, place, dependence, physiological	Entertainment, escapism, esteem, self-actualization

**Table 2:** Preliminary evaluation indicators for CCTPD.

Measurement elements	Sub-items
Sense experience design	S1 Aesthetics: The colors and shapes meet my aesthetic needs.
	S2 Creativity: Exhibits a unique sense of creativity that makes me feel novel.
	S3 Cultural traits: Reflects cultural/regional characteristics effectively.
	S4 Authenticity: Restores and reflects the inherent attributes of the culture effectively.
	S5 Craft: Effectively uses craft, materials, and textures.
Act experience design	A1 Usability: The usage process is simple and convenient, and it meets my ergonomic requirements.
	A2 Functionality: Provides me with a good operational experience.
	A3 Interactivity: Provides me with a good interactive experience.
	A4 Participation: Provides me with many opportunities to participate and immerse myself in the culture.
Think experience design	T1 Memorability: Reminds me of the cultural experience.
	T2 Appeal: It attracts me and makes me interested in learning about the culture.
	T3 Educational value: Helps me understand and disseminate the culture more effectively.
	T4 Thought-provoking: The cultural connotations it conveys help me think creatively and possess a certain degree of spiritual value.
Relate experience design	R1 Shareable: Makes me willing to share it on social media or display it to others to highlight my taste.
	R2: Social Perception: Makes me feel that I belong to the cultural circle.
	R3 Sociability: Helps me socialize and find like-minded friends.
	R4 Cost-effectiveness: Provides me with a satisfying consumption experience.
Feel experience design	F1 Fun: Makes me feel that it is fun and interesting.
	F2 Fashion trend: Conforms to modern trends and fashion.
	F3 Escapism: Provides me with an immersive experience that makes me temporarily forget my original life.
	F4 Emotional resonance: Elicits emotional resonance.
	F5 Emotional healing: Provides me with some degree of emotional healing.

#### 4.2.2 Measurement items

To measure the variables described in Figure 1, the scales used in this study were based on previous research published in authoritative journals. An initial survey questionnaire was developed through a review and analysis of the relevant literature. The measurement items from the reviewed literature were coded and analyzed, and the KJ method was employed to classify and name the eight main measurement elements and 31 sub-elements. Five research experts familiar with the current topic area were invited to review the questionnaire. Measurement elements with high consistency were selected, and descriptions of certain indicators were revised. Based on their feedback regarding wording, phrasing, and layout, minor modifications were made. Once no further revisions were requested, the complete initial survey questionnaire for this study was finalized.

To refine the formal questionnaire, we conducted a pretest by distributing 100 questionnaires at the Chongqing Three Gorges Museum and collecting 91 valid responses. This pretest provided preliminary evidence of reliability and helped streamline the instrument to keep it concise. An exploratory factor analysis (EFA) was conducted to examine the factor structure and identify poorly performing items. Based on the pretest results, four items (SED5, TED1, RED4, FED2) were removed because their factor loadings were below 0.60, and the remaining items were retained for the final survey. The questionnaire consisted of two parts: formal measurement items and demographic information. All items were scored on a 7-point Likert scale (1 = strongly disagree, 7 = strongly agree). Given the potential for response clustering at the upper end of the scale, we examined item distributions (means, standard deviations, skewness/kurtosis, and the proportion of maximum scores) to assess possible ceiling effects. We estimated indirect effects using bootstrap-based confidence intervals to evaluate the robustness of mediation under potential non-normality and limited variance. Demographic information included gender, age, education level, monthly income, and occupation. The formal measurement items captured the psychological generation mechanism of cultural confidence (cultural cognition, cultural identity, cultural confidence) and CCTPD across five experiential dimensions (sense, act, think, relate, and feel). Internal consistency reliability was evaluated using Cronbach’s alpha (see Table 3).

**Table 3:** Construct measurement items and measurement-model results.

Items	Factor loading	Cronbach’s Alpha	CR	AVE
*Cultural Cognition (CG)*		0.783	0.783	0.547
CG1: Increased my understanding of Chinese cultural heritage (e.g., clothing, crafts, or buildings).	0.731			
CG2: Increased my understanding of Chinese historical stories (e.g., historical figures, dynastic changes, or legends).	0.735			
CG3: Increased my understanding of Chinese customs and traditions (e.g., traditional festivals or minority cultures).	0.753			
*Cultural Identity (CI)*		0.856	0.862	0.611
CI1: Made me believe that it is very important to maintain my Chinese culture.	0.804			
CI2: Although I might acquire some elements of another culture(s), it is important for me to maintain my Chinese culture.	0.810			
CI3: Made me believe that it is very important for children to learn the values associated with Chinese culture.	0.832			
CI4: Made me believe that participating in Chinese cultural festivals and activities is important to me.	0.669			
*Cultural Confidence (CC)*		0.833	0.836	0.562
CC1: Convinced me that Chinese culture is unique with respect to other cultures.	0.783			
CC2: Chinese culture can help me think about and solve problems that I encounter.	0.804			
CC3: Although Chinese culture may have shortcomings or flaws, that fact does not affect my recognition of it.	0.745			
CC4: Made me feel proud of and confident in Chinese culture.	0.657			
*CCTPD 1: Sense Experience (SE)*		0.827	0.828	0.671
S1 Aesthetics: The colors and shapes meet my aesthetic needs.	0.736			
S2 Cultural traits: Reflects cultural/regional characteristics effectively.	0.793			
S3 Authenticity: Restores and reflects the inherent attributes of the culture effectively.	0.826			
*CCTPD 2: Act Experience (AE)*		0.810	0.810	0.588
A1 Usability: The usage process is simple and convenient, and it meets my ergonomic requirements.	0.735			
A2 Interactivity: Provides me with a good interactive experience.	0.752			
A3 Participation: Provides many opportunities for me to participate and immerse myself in the culture.	0.810			
*CCTPD 3: Think Experience (TE)*		0.844	0.846	0.648
T1 Educational value: Helps me understand and disseminate the culture more effectively.	0.815			
T2 Thought-provoking: The cultural connotations it conveys help me think creatively and possess a certain degree of spiritual value.	0.807			
T3 Attractiveness: Attracts me and makes me interested in learning about the culture.	0.790			
*CCTPD 4: Relate Experience (RE)*		0.788	0.801	0.574
R1 Shareability: Makes me willing to share it on social media or display it to others to highlight my taste.	0.793			
R2 Social perception: Makes me feel that I belong to the cultural circle.	0.808			
R3 Sociability: Helps me socialize and find like-minded friends.	0.664			
*CCTPD 5: Feel Experience (FE)*		0.854	0.855	0.597
F1 Emotional healing: Provides me with some degree of emotional healing.	0.767			
F2 Emotional resonance: Elicits emotional resonance.	0.797			
F3 Escapism: Provides me with an immersive experience that makes me temporarily forget my original life.	0.774			
F4 Fun: Makes me feel that it is fun and interesting.	0.752			

##### 4.2.2.1 Cultural cognition

The measurement items for cultural cognition were based on the cultural cognition elements proposed by Yang et al. (2022) and the cultural creative tourism design principles suggested by Song et al. (2021), drawing on cognitive psychology. These items were designed in consideration of the actual context of the Three Gorges Museum’s cultural creative experience pavilion and the objectives of this study. The measurement items encompassed three dimensions —cultural heritage, historical stories, and folk customs —to more accurately reflect visitors’ cultural cognition during the cultural creative experience. An example item was: “Increased my understanding of Chinese cultural heritage (e.g., clothing, crafts, or buildings).”

##### 4.2.2.2 Cultural identity

The measurement items for cultural identity were primarily based on the study by Cleveland and Bartikowski (2018), which adapted ethnic identity to the context of Chinese culture. This measurement framework had been widely used internationally. The questions were restructured around our research theme, covering four aspects: social interaction, participation in ethnic customs and celebrations, the importance of traditional values and norms, and self-identity and pride. An example item was: “I consider it very important to maintain my Chinese culture.”

##### 4.2.2.3 Cultural confidence

The measurement items for cultural confidence were adapted from the cultural confidence scales proposed by Pan et al. (2021) and Zhou and Bi (2020), which were widely applied in the context of Chinese cultural confidence. Pan et al.’s scale, from a psychological perspective, included two dimensions: self-confidence and efficacy. In contrast, Zhou and Bi′s scale included cultural praise and cultural pride, with college students as the validation sample. This study made appropriate modifications to ensure accurate measurement of the cultural confidence generated by visitors during their participation in the cultural product experience. An example item was: “Chinese culture can help me think about and solve problems that I encounter.”

##### 4.2.2.4 CCTPD

The measurement items for the experiential dimensions of CCTPD were based on the preliminary evaluation indicators developed earlier. The scale included five dimensions—sense experience, act experience, think experience, relate experience, and feel experience—comprising 16 items in total. An example item was: “The cultural connotations it conveys help me think creatively and possess a certain degree of spiritual value. “.

### 4.3 Data collection and sample

The survey was conducted from April 15, 2024, to April 24, 2024, at the Cultural and Creative Experience Pavilion at the Three Gorges Museum in Chongqing, using a convenience sampling method. Visitors were interviewed at the conclusion of their tours. The survey was administered by eight research assistants who had received one training session in the data collection process. The interviews lasted 10 min on average and were audio-recorded after obtaining the visitors’ consent. Participants could withdraw from the study at any time, and those who completed the entire questionnaire received a small gift. The main reason for choosing a convenience sampling method in this study is that the visitor population at the Three Gorges Museum Cultural Creative Experience Pavilion is highly mobile and widely distributed. Convenience sampling is cost-effective when time and resources are limited, making it suitable for exploratory research (Andrade, 2021). To minimize biases in data collection, several measures were taken: first, during the survey design phase, expert reviews and pre-surveys were conducted to ensure the questionnaire’s scientific validity. Second, all surveyors received professional training to ensure standardized data collection. Finally, sampling was conducted across different time periods and visitor groups to cover a diverse range of backgrounds and reduce sample bias. Data collection transparency was ensured to reduce bias. In the end, 325 questionnaires were distributed, and 314 were collected as valid. To ensure an adequate sample size for reliable modelling, this study conducted a *post hoc* power analysis. The results indicated that the statistical power for each endogenous variable was high (greater than 0.80) (Cohen, 1988). Therefore, the sample size used in this study (*n* = 314) possesses sufficient statistical power to support the SEM results.

### 4.4 Data analysis strategy

Structural equation modelling (SEM) was conducted in IBM SPSS Amos 24.0 to evaluate the succession of dependent relationships and to validate the relationships among the multiple independent and dependent constructs included in this research. To assess the conceptual model, a two-step procedure was employed. First, the measurement model—specified with reflective constructs—was assessed for reliability and validity using composite reliability (CR), Cronbach’s alpha, standardized factor loadings, and average variance extracted (AVE). After establishing the quality of the measurement scales, the structural model was estimated to clarify the relationships among the latent variables.

### 4.5 Common method bias

Because the data were collected using a self-report questionnaire in a single survey, we assessed potential common method bias. In addition to procedural remedies during questionnaire design and administration (e.g., pretest-based item refinement, standardized instructions, and trained survey administrators), we conducted Harman’s single-factor test by entering all measurement items into an unrotated EFA. If a single factor accounts for the majority of the total variance (commonly >50%), common method bias may be a concern.

## 5 Results

To examine the proposed model, we analyzed questionnaire data collected from visitors to a cultural site and tested the associations among CCTPD, cultural cognition, cultural identity, and cultural confidence. Specifically, we assessed whether CCTPD and its experiential dimensions were related to visitors’ cultural cognition, cultural identity, and cultural confidence, and whether the data were consistent with the hypothesized sequential mediation pattern.

Common method bias check. Harman’s single-factor test showed that the first factor accounted for 40.551% of the total variance, which is below the commonly used threshold of 50%, suggesting that common method bias was unlikely to be a substantial concern in this study.

### 5.1 Profile of the respondents

The demographic profile of the sample is presented in Table 4. Fewer male respondents (37.3%) than female respondents (62.7%) completed the survey. Nearly half of the respondents (43.3%) were aged 18–24, and the next most common age groups were 25–34 (28.0%) and 35–44 (16.9%). Most of the respondents had a bachelor’s degree (69.7%), and the second most common level of education was a master’s degree (20.4%). With respect to respondents’ monthly income, the group of respondents earning less than ¥2,500 was the largest (37.6%), followed by the other groups, such as those earning ¥2,501–5,000 (17.2%), ¥7,501–10,000 (16.6%) and ¥5,001–7,500 (14.6%), which accounted for similar portions of the sample. In terms of respondents’ occupations, most of the respondents were students (40.8%) or staff members working for enterprises or public institutions (36.0%); the remaining respondents could be divided approximately equally into three types: laborers (6.1%), businesspersons (3.1%), and farmers (1.9%).

**Table 4:** Demographic profile of the sample.

Variables	Frequency (%)
Gender	
Male	117 (37.3%)
Female	197 (62.7%)
Age	
18–24	136 (43.3%)
25–34	88 (28.0%)
35–44	53 (16.9%)
45–54	26 (8.3%)
> = 55	11 (3.5%)
Level of education	
High school and lower	11 (3.5%)
High school education	20 (6.4%)
Bachelor’s degree	219 (69.7%)
Master’s degree	64 (20.4%)
Monthly income	
<= ¥2,500	118 (37.6%)
¥2,501–5,000	54 (17.2%)
¥5,001–7,500	46 (14.6%)
¥7,501–10,000	52 (16.6%)
<=¥10,000	44 (14.0%)
Occupation	
Laborer	19 (6.1%)
Farmer	6 (1.9%)
Businessperson	10 (3.1%)
Staff member working for an enterprise or public institution	113 (36.0%)
Student	128 (40.8%)
Other	38 (12.1%)

### 5.2 Model assessment

**Figure 2:**
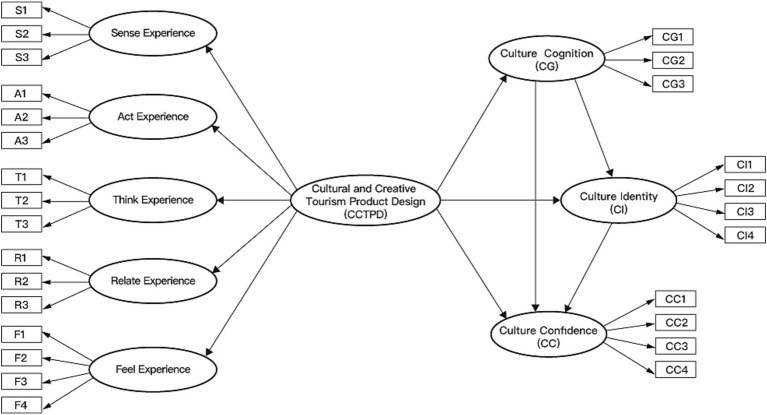
Model 1: higher-order CCTPD (sense/act/think/relate/feel) → cultural cognition → cultural identity → cultural confidence (SEM, *N* = 314).

**Figure 3:**
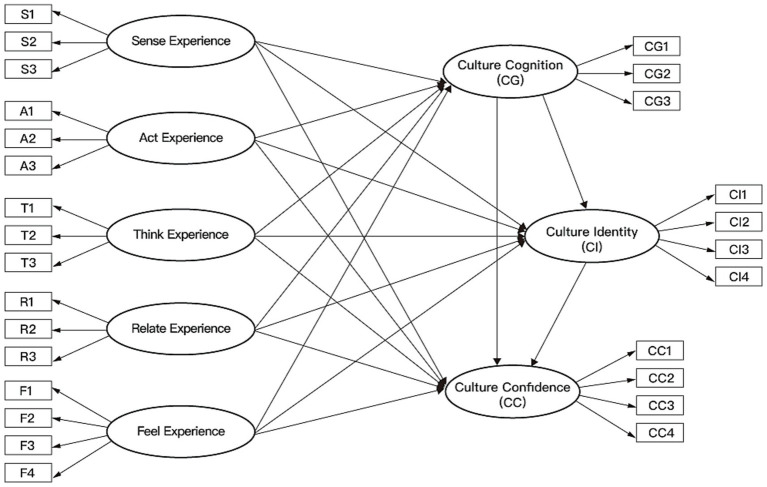
Model 2: five CCTPD dimensions and their differentiated paths to cultural cognition, identity, and confidence (SEM, *N* = 314).

To explore the overall relationship between CCTPD and the psychological generation mechanism of cultural confidence, this study treated the five experiential dimensions of CCTPD as first-order variables. A single overall latent variable representing CCTPD is extracted as a second-order variable to construct Model 1, as shown in Figure 2. The final model consists of 9 constructs. Furthermore, to investigate the specific impact paths between the different dimensions of CCTPD and the various stages of cultural confidence generation, as shown in Model 2 in Figure 3, this study developed an 8-variable model to examine the direct effects of the five dimensions on cultural cognition, cultural identity, and cultural confidence.

#### 5.2.1 Assessment of the measurement model

Because the data were collected only once from the respondents, all variables were estimated from the participants’ perspective, and the data were collected from the same participants (i.e., visitors to the Cultural and Creative Experience Pavilion at the Three Gorges Museum). Common method bias (CMV) may thus have been an issue in this research. Although the study was conducted at multiple time points (i.e., a two-stage survey) and employed anonymous measurement and item cross-arrangement methods to mitigate CMV, it was necessary to conduct additional statistical tests to assess CMV. Thus, this study compared models based on the initial prespecified model using the latent-error-variable control method. In the hypothesized model (an eight-factor model), CMV was included as a latent variable. The test revealed that the model without CMV exhibited no significant differences from the model with CMV (χ^2^/df = 1.38, GFI = 0.919, AGFI = 0.887, RMSEA = 0.035, CFI = 0.977, TLI = 0.97) and that ΔCFI = 0.008 < 0.05, thus indicating that CMV did not significantly affect the relationships included in the model (Bagozzi and Yi, 1988).

To assess the reliability of the models, this study conducted confirmatory factor analysis (CFA) to investigate all the constructs included in the models. As shown in Tables 3, 5, 6, the Cronbach’s *α* coefficients for each construct in both models exceeded 0.70, thus indicating acceptable reliability. The factor loadings for each indicator on its corresponding latent variable were calculated. Except for three indicators that exhibited factor loadings ranging between 0.6 and 0.7, all other items exhibited factor loadings that were above the acceptable threshold (i.e., greater than 0.7) (Hair et al., 2011). The composite reliability (CR) values exceeded the recommended threshold of 0.7, the average variance extracted (AVE) values for each construct exceeded the 0.5 threshold (Fornell and Larcker, 1981), and the square roots of the AVE values were greater than the correlations among the constructs (Martynova et al., 2018). These results indicated that the models exhibited high discriminant validity and acceptable levels of internal consistency reliability and convergent validity. Given that the CR and AVE estimates exceeded the thresholds, removing indicators with factor loadings below 0.7 from the models was unnecessary. These results confirmed the acceptable reliability of the measurement models used in this research.

**Table 5:** The discriminant validity of the constructs included in Model 1.

Constructs	AVE	CCTPD	CG	CI	CC
CCTPD	0.652	**0.807**			
CG	0.547	0.714	**0.739**		
CI	0.611	0.663	0.608	**0.781**	
CC	0.562	0.742	0.534	0.736	**0.749**

Bold diagonal entries are the square roots of AVE.

**Table 6:** The discriminant validity of the constructs included in Model 2.

Constructs	AVE	FE	RE	TE	AE	SE	CG	CI	CC
FE	0.597	**0.773**							
RE	0.574	0.692	**0.758**						
TE	0.648	0.647	0.606	**0.804**					
AE	0.588	0.654	0.556	0.721	**0.766**				
SE	0.671	0.675	0.530	0.661	0.747	**0.786**			
CG	0.547	0.507	0.428	0.610	0.664	0.627	**0.740**		
CI	0.611	0.488	0.524	0.600	0.562	0.510	0.608	**0.781**	
CC	0.562	0.642	0.630	0.594	0.550	0.609	0.535	0.736	**0.749**

Bold diagonal entries are the square roots of AVE.

#### 5.2.2 Assessment of the structural model

This study used the discriminant validity method recommended by Hair (2011) for the two models, as shown in Table 7; the chi-square-to-degrees-of-freedom ratios were 1.471 and 1.557, respectively. Model 1 exhibited an RMSEA of 0.039, and Model 2 exhibited an RMSEA of 0.042; both values were below the 0.05 threshold and thus met the fit index standards, indicating a close fit of the model in relation to the degrees of freedom and suggesting that the model represents the actual data well (Hair, 2011). AGFI and CFI values greater than 0.9 generally indicate a good fit. Except for the adjusted goodness-of-fit index (AGFI) of Model 1 and the AGFI and goodness-of-fit index (GFI) of Model 2, which were slightly below 0.9 (but within acceptable ranges; Hair, 2011), all the indices met the relevant standards. Additionally, the NFI values for Model 1 and Model 2 were 0.909 and 0.898, respectively, which were close to 0.9 and still considered a good fit in complex models or large-sample contexts (Wu et al., 2024). Moreover, the TLI values for both models were greater than 0.9, indicating a good fit relative to the baseline model (Ramlall, 2016). The SRMR values were 0.0391 for Model 1 and 0.0464 for Model 2, both below the recommended cutoff (e.g., SRMR <0.08), indicating an acceptable fit (Hu and Bentler, 1999). The results showed that the model’s overall indicators were at an acceptable level.

**Table 7:** Results regarding the model fit measures.

Model	χ^2^/df	GFI	AGFI	RMSEA	CFI	NFI	TLI	SRMR
Model 1	1.471	0.906	0.880	0.039	0.969	0.909	0.963	0.0391
Model 2	1.557	0.894	0.872	0.042	0.961	0.898	0.956	0.0464

### 5.3 Hypothesis tests

#### 5.3.1 The impact of CCTPD on the psychological generation of cultural confidence

**Figure 4:**
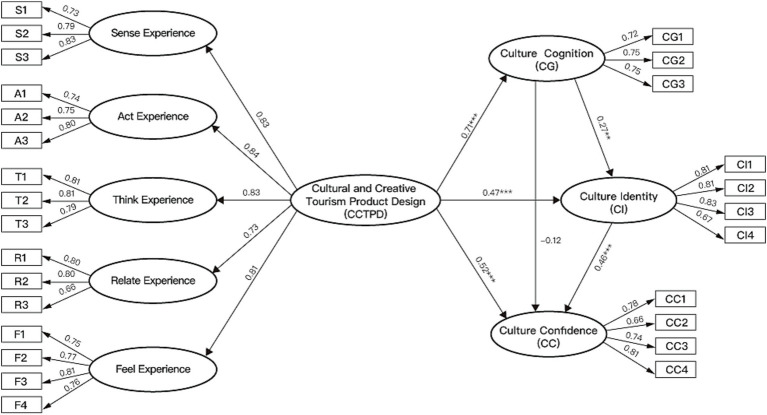
The result of Model 1 (structural paths and standardized estimate).

Five of the six hypotheses were supported (see Table 8; Figure 4). Cultural cognition had a significant positive effect on cultural identity (*β* = 0.275, *t*-value = 2.934, *p* < 0.01), but not on cultural confidence (*β* = −0.119, *t*-value = −1.357, *p* > 0.05). Cultural identity had a significant positive effect on cultural confidence (*β* = 0.464, *t*-value = 5.995, *p* < 0.001). Thus, H1 and H3 were supported, while H2 was rejected. CCTPD, as hypothesized, had significant positive effects on cultural cognition (*β* = 0.714, *t*-value = 9.04, *p* < 0.001), cultural identity (*β* = 0.467, *t*-value = 4.936, *p* < 0.001), and cultural confidence (*β* = 0.519, *t*-value = 5.412, *p* < 0.001). Thus, H4, H5 and H6 were supported.

**Table 8:** Results of the hypothesis tests.

Hypothesis	Relationships	Std.	Estimate	S.E.	*T*-value	*p*	Test result
H1	CG → CI	0.275	0.292	0.100	2.934	**	Yes
H2	CG → CC	−0.119	−0.127	0.094	−1.357	0.175	No
H3	CI → CC	0.464	0.469	0.078	5.995	***	Yes
H4	CCTPD→CG	0.714	0.685	0.076	9.040	***	Yes
H5	CCTPD→CI	0.467	0.477	0.097	4.936	***	Yes
H6	CCTPD→CC	0.519	0.536	0.099	5.412	***	Yes

*p < 0.05, **p < 0.01, ***p < 0.001.

The effect of cultural cognition on cultural confidence was not significant, and cultural identity occupied an intermediate position between the two paths linking cultural cognition to cultural confidence and CCTPD to cultural confidence. Therefore, it is necessary to explore the mediating effect of cultural identity to improve the understanding of the relationships among cultural cognition, CCTPD, and cultural confidence (see Table 9). To investigate the mediating effect, the direct, indirect, and total effects between the variables were measured using the bootstrap method, which does not require the assumption of a normal data distribution. This method is particularly suitable for analyzing indirect effects, providing more accurate confidence intervals. In this study, 5,000 bootstrap resamples were used to calculate 95% confidence intervals (CI). A confidence interval that does not include 0 indicates a statistically significant effect.

**Table 9:** Mediation tests: cultural cognition and cultural identity as mediators (5,000-sample bootstrap; 95% CI).

Effect path		Point estimate	Product of coefficients		Bias-corrected percentile 95%CI		Mediation
			S.E.	Z-value	Lower	Upper	
CG → CC	Total effects	0.570	0.096	5.938	0.402	0.774	Yes
	Direct effects	0.134	0.106	1.264	−0.080	0.345	
CG → CI → CC	Indirect effects	0.435	0.098	4.439	0.279	0.665	
CCTPD→CC	Total effects	0.766	0.087	8.805	0.611	0.957	Yes
	Direct effects	0.536	0.117	4.581	0.326	0.788	
CCTPD→CG → CI → CC	Indirect effects of CG	−0.087	0.073	−1.192	−0.245	0.046	
	Indirect effects of CI	0.223	0.077	2.896	0.101	0.401	
	Indirect effects of CG&CI	0.094	0.042	2.238	0.034	0.201	

Firstly, this study identified whether the relationship between cultural cognition and cultural confidence was mediated by cultural identity. The indirect effect of cultural cognition on cultural confidence via cultural identity (*β* CG-CI-CC = 0.435, 95% CI: 0.279–0.665) was significant and positive, while the direct effect of cultural cognition on cultural confidence (*β* CG-CC = 0.134, 95% CI: −0.080–0.345) was not significant. This indicates that cultural identity fully mediates the relationship between cultural cognition and cultural confidence, suggesting that cultural cognition does not directly influence cultural confidence but must exert its effect through cultural identity.

Next, this study explored the psychological mechanism underlying the formation of cultural confidence in the context of cultural and creative tourism products. The results of the path analysis revealed that CCTPD had a significant total effect on cultural confidence (*β* = 0.766, 95% CI: 0.611–0.957). Additionally, the direct effect of CCTPD on cultural confidence was also significant (*β* CCTPD-CC = 0.536, 95% CI: 0.326–0.788). Regarding the mediating effects, cultural cognition and cultural identity played varying roles in the relationship between CCTPD and cultural confidence. Specifically, the indirect effect of cultural cognition was not significant (*β* CCTPD-CG-CC = −0.087, 95% CI: −0.245, 0.046), indicating that CCTPD’s impact on cultural confidence was not mediated through cultural cognition alone. In contrast, the indirect effect of cultural identity was significant (*β* CCTPD-CI-CC = 0.223, 95% CI: 0.101–0.401), suggesting that cultural identity served as a crucial mediator in this relationship. Moreover, the chain mediation effect through cultural cognition and cultural identity was also significant (*β* CCTPD-CG-CI-CC = 0.094, 95% CI: 0.034–0.201), implying that CCTPD indirectly influenced cultural confidence through cultural cognition, which subsequently affected cultural identity.

In summary, the psychological mechanism of cultural confidence formation highlights the full mediating role of cultural identity as a central component. Cultural cognition must be transformed through cultural identity to influence cultural confidence. Within the impact of CCTPD on the psychology of cultural confidence, cultural cognition is not a necessary condition for triggering cultural confidence. Compared to cultural cognition, cultural identity plays a more critical role in both the chain and single-path mediation processes.

#### 5.3.2 The influence of specific dimensions of CCTPD on cultural cognition, cultural identity, and cultural confidence

In the direct effects of cultural and creative tourism products, this study has confirmed that their experience design can significantly impact cultural cognition, cultural identity, and cultural confidence. Considering that different stages may involve different influencing mechanisms, this study further explored the role dimensions at each stage.

**Figure 5:**
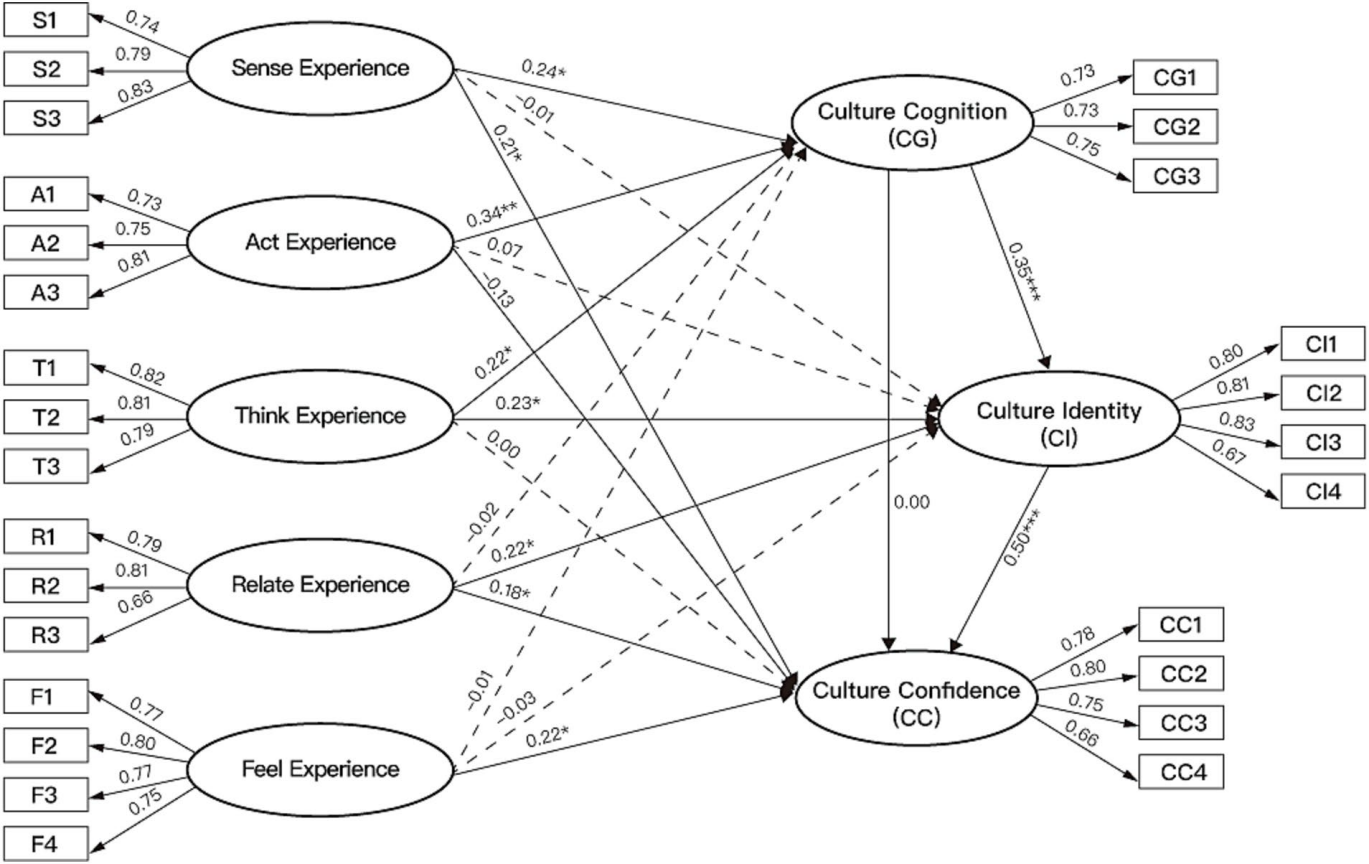
Standardized paths for Model 2 by CCTPD dimensions.

As shown in Table 10 and Figure 5, in the stage of cultural cognition, sense experience (*β* = 0.242, t-value = 2.161, *p* < 0.05), act experience (*β* = 0.339, *t*-value = 2.697, *p* < 0.01), and think experience (*β* = 0.221, *t*-value = 2.118, *p* < 0.05) were observed to have significant direct relationships with cultural cognition. However, the effects of relate experience (*β* = −0.016, *t*-value = −0.167, *p* > 0.05) and feel experience (direct effect = −0.008, *t*-value = −0.09, *p* > 0.05) on cultural cognition were shown to be not significant. In the stage of cultural identity, think experience (*β* = 0.226, *t*-value = 2.270, *p* < 0.05) and relate experience (*β* = 0.224, *t*-value = 2.462, *p* < 0.05) were observed to have significant direct relationships with cultural identity. However, the effects of sense experience (*β* = −0.007, *t*-value = −0.067, *p* > 0.05), act experience (*β* = 0.068, *t*-value = 0.579, *p* > 0.05), and feel experience (*β* = −0.029, *t*-value = −0.287, *p* > 0.05) on cultural identity were shown to be not significant. As for cultural confidence, the results indicated that sense experience (*β* = 0.209, *t*-value = 2.189, *p* < 0.05), relate experience (*β* = 0.177, *t*-value = 2.167, *p* < 0.05), and feel experience (*β* = 0.217, *t*-value = 2.373, *p* < 0.05) were shown to have significant direct relationships with cultural confidence. However, the effects of act experience (*β* = −0.127, *t*-value = −1.198, *p* > 0.05) and think experience (*β* = 0.000, *t*-value = 0.000, *p* > 0.05) on cultural confidence were not significant.

**Table 10:** Effects of specific CCTPD dimensions at each psychological stage (cognition/identity/confidence).

Stage	Relationships	Std.	Estimate	S.E.	*T*-value	*p*	Test result
Cultural cognition	SE → CG	0.242	0.204	0.095	2.161	*	Yes
	AE → CG	0.339	0.272	0.101	2.697	**	Yes
	TE → CG	0.221	0.204	0.097	2.118	*	Yes
	RE → CG	−0.016	−0.014	0.084	−0.167	0.867	No
	FE → CG	−0.008	−0.008	0.090	−0.090	0.928	No
Cultural identity	SE → CI	−0.007	−0.006	0.094	−0.067	0.946	No
	AE → CI	0.068	0.058	0.099	0.579	0.563	No
	TE → CI	0.226	0.219	0.096	2.270	*	Yes
	RE → CI	0.224	0.209	0.085	2.462	*	Yes
	FE → CI	−0.029	−0.025	0.088	−0.287	0.774	No
Cultural confidence	SE → CC	0.209	0.187	0.086	2.189	*	Yes
	AE → CC	−0.127	−0.108	0.090	−1.198	0.231	No
	TE → CC	0.000	0.000	0.088	−0.001	0.999	No
	RE → CC	0.177	0.168	0.078	2.167	*	Yes
	FE → CC	0.217	0.191	0.080	2.373	*	Yes

*p < 0.05, **p < 0.01, ***p < 0.001.

## 6 Discussion

This study used the Cultural and Creative Experience Pavilion at the Chongqing Three Gorges Museum (China) as a case to examine the associations among CCTPD and its experiential dimensions, cultural cognition, cultural identity, and cultural confidence. Using SEM, the results support the proposed model in which CCTPD is positively associated with cultural confidence, with cultural identity functioning as a key mediator in the hypothesized pathways.

Overall, the findings are consistent with a cognition–identity–confidence pathway, suggesting that experience design attributes may contribute to visitors’ cultural confidence through cognitive and identity-related processes. However, given the cross-sectional design, these relationships should be interpreted as associations rather than causal effects.

### 6.1 The crucial role of cultural identity in the psychological generation of cultural confidence

Understanding the psychological formation of cultural confidence remains an important research topic. Although some scholars have suggested a direct link between cultural cognition and cultural confidence, our results indicate that cultural identity fully mediates the association between cultural cognition and cultural confidence. Specifically, cultural cognition was not significantly associated with cultural confidence when cultural identity was included, whereas cultural identity showed a strong association with cultural confidence. These findings are consistent with arguments by Guan (2023) and Wang and Huang (2018) that cultural cognition provides a basis for cultural identity, while cultural confidence often involves an additional process of psychological identification. In this view, cultural cognition may provide individuals with a basic understanding of culture, but the accumulation of cognitive information alone may be insufficient to correspond to higher cultural confidence. If cognitive understanding does not extend to emotional and value-based recognition, it may be less likely to coincide with stronger cultural confidence. Without such identification, confidence in culture may remain relatively superficial (Lin et al., 2022) or may even be expressed as “blind confidence” (Wang and Huang, 2018).

Moreover, the internalization of cultural cognition is often gradual and multifaceted. Cognitive processing can be planned, intentional, and reflective, suggesting that internalization is not only information processing but also a goal-directed psychological activity (Rosenbaum, 2001). Cultural confidence may be more likely to co-occur when cultural cognition aligns with individuals’ values and social identity, supporting the formation of recognition on a cognitive basis and, in turn, being associated with higher cultural confidence.

This perspective may also help explain why our findings differ from those reported by Zhou et al. (2022). Zhou et al. used Chinese “red culture” as the cognitive carrier, which often evokes national pride and identity-relevant meanings; thus, cultural cognition in that context may be more strongly linked to cultural confidence. By contrast, the cultural carrier in our study was historical culture, and visitors’ cognition was largely derived from cultural and creative tourism products or indirect and fragmented information. In this context, visitors’ cultural cognition may be more oriented toward factual learning, which may be less directly associated with cultural confidence unless accompanied by identity-related appraisal and emotional resonance.

### 6.2 CCTPD and the psychological formation of cultural confidence: multiple pathways

First, this study examined the associations between CCTPD and different psychological stages related to cultural confidence. The SEM results indicated that CCTPD was positively associated with cultural cognition, cultural identity, and cultural confidence, with relatively large standardized coefficients (*β* > 0.5) for these paths. Consistent with prior work, these associations are in line with the idea that cultural and creative tourism products may correspond to multiple experience functions, including cognitive engagement (Song et al., 2021), social support (Wang and Yang, 2023), and affective experience (Feng et al., 2023). In this perspective, individuals may acquire cultural knowledge through cognitively oriented experiences, develop a sense of belonging through identity-related appraisal, and experience pride through cultural comparison. Together, these patterns help contextualize the multiple pathways linking cultural and creative tourism products with cultural confidence and are broadly consistent with interpretations informed by information-processing theory, social identity theory, and cultural comparison mechanisms in tourism-related cultural creativity.

Interestingly, the standardized association between CCTPD and cultural cognition was the largest (*β* = 0.714, *p* < 0.001), exceeding those with cultural confidence (*β* = 0.519, *p* < 0.001) and cultural identity (*β* = 0.467, *p* < 0.001). One possible interpretation is that cultural and creative tourism products may be particularly effective in communicating material or behavioral culture (Yang et al., 2022). Observations from the Cultural and Creative Experience Pavilion at the Three Gorges Museum also suggest that product design often emphasizes the extraction and transformation of cultural elements, which may be more immediately accessible to visitors and therefore more strongly linked to cultural cognition. In this sense, material and behavioral aspects of culture may be more readily perceived than more abstract spiritual culture.

Regarding indirect effects, cultural cognition and cultural identity appeared to play different roles in the association between CCTPD and cultural confidence. The specific indirect effect via cultural cognition alone was not significant, suggesting that the association between CCTPD and cultural confidence is unlikely to operate solely through increased cultural cognition. This may be because cultural cognition primarily reflects knowledge acquisition and informational understanding, whereas cultural confidence may require additional affective and value-based recognition. Thus, transmitting cultural knowledge through cultural and creative tourism products may not be sufficient to correspond to higher cultural confidence without identity-relevant and emotional components. In contrast, cultural identity showed a significant indirect effect between CCTPD and cultural confidence, indicating that when product experiences are associated with deeper recognition of cultural values, the relationship with cultural confidence tends to be stronger (Feng et al., 2023). This pattern suggests that cultural and creative tourism products may relate to cultural confidence partly by being associated with stronger feelings of cultural belonging and identification.

Additionally, we observed a significant sequential indirect effect through cultural cognition and cultural identity, consistent with a pathway in which CCTPD is associated with cultural cognition, which is then associated with cultural identity, and subsequently with cultural confidence. This sequential pattern highlights the potential interplay between cognitive and affective processes in cultural communication: knowledge-oriented content may be more likely to translate into cultural confidence when it co-occurs with identity-related appraisal and emotional resonance.

### 6.3 Dimension-specific patterns linking CCTPD to cultural cognition, cultural identity, and cultural confidence

#### 6.3.1 SENSE, ACT, and THINK experience design are positively associated with cultural cognition

Sense experience emphasizes the intuitive communication of culture through design elements such as patterns, colors, and materials, which may help visitors access and interpret cultural meanings—particularly more tangible aspects of culture. This direct, sensory-oriented approach may be linked to greater initial interest and preliminary cultural understanding, which aligns with Zhou and Han (2024). From the perspective of cultural hierarchy theory, the “external” level of culture—including visible and material elements—tends to be more readily communicated and recognized (Yang et al., 2022).

Act experience highlights participation and interaction during the visitor experience. Act-oriented design often aligns with tourism consumers’ everyday lifestyles and may incorporate persuasive elements (Yang, 2022). For example, in the Cultural and Creative Experience Pavilion of the Three Gorges Museum, visitors may take part in hands-on activities such as making round fans or seals, allowing them to experience aspects of intangible cultural heritage craftsmanship. Compared with merely watching or reading, such participatory activities may encourage deeper engagement and exploration, which in turn may relate to stronger cultural understanding. This kind of experience may also be closer to everyday cognition and may facilitate the internalization of cultural information, thereby being positively associated with cultural cognition. In a similar vein, Zhao and Duan (2024) reported that improvements in virtual interactive experiences for non-traditional culture were associated with higher levels of tourists’ cultural cognition.

Think experience involves cognitive processes such as memory, perception, imagination, and language. Consistent with Jiang et al. (2024), cultural and creative tourism products may invite visitors to reflect on cultural information through intellectually stimulating and creative designs, such as interactive Q&A, decoding booklets, or archaeology-themed blind boxes. In this way, visitors are not only passive recipients of information; they may also engage in active reflection to connect new knowledge with existing understanding. Think-oriented experiences may therefore be linked to deeper processing of cultural information and stronger memory through analysis and association, which corresponds to higher cultural cognition.

By contrast, feel and relate experiences were not significantly associated with cultural cognition in our model. One possible explanation relates to the functional focus of these experience types. Feel experience is more oriented toward emotional responses and inner resonance, emphasizing affective-level reactions and emotional connection to culture (Ji and Li, 2025). Relate experience focuses more on collective experience and social interaction than on acquiring cultural knowledge. During shared experiences, visitors may attend more to participation and sharing; although such processes can support cultural identity, they may be less directly tied to learning-oriented cultural cognition.

Overall, during the cultural cognition stage, CCTPD appears to be primarily linked to learning-oriented engagement, but the ways in which experience dimensions relate to cognition differ. Sense, act, and think experiences correspond to sensory contact, embodied participation, and cognitive stimulation, respectively, and are positively associated with visitors’ acquisition, processing, and understanding of cultural information. From a design perspective, sensory-oriented design may integrate cultural elements through appropriate choices of color, materials, and surface treatments; act-oriented design may connect interactive methods to everyday behaviors to support embodied engagement with cultural characteristics; and think-oriented design may popularize cultural knowledge and encourage reflection through prompts that elicit interpretation and meaning-making. Although relate and feel experiences are more strongly aligned with social and emotional needs, this does not imply that they are irrelevant to cultural cognition. Future work could explore how emotional engagement and social interaction may indirectly support learning (e.g., by increasing motivation and attention). For instance, museums might create spaces for cultural exchange among visitors or between creators and visitors, or employ technologies such as VR/AR to enhance interest and immersion, potentially supporting cultural cognition through multiple pathways.

#### 6.3.2 THINK and RELATE experience design are positively associated with cultural identity

Think experience can be understood as a cognitively oriented process in which visitors reflect on and learn from cultural content. Such reflection and learning may provide an informational and meaning-based foundation for cultural identity formation. Moreover, when cultural knowledge resonates with visitors’ personal values during reflection, it may be accompanied by emotional resonance (Tian et al., 2020), which is often linked to stronger identification with cultural meanings.

In this study, relate experience emphasizes sharing and social interaction, meaning that visitors may engage in cultural activities with others or share their experiences. Social influence theory suggests that individuals’ attitudes, beliefs, and behaviors may shift in response to others or reference groups (Kelman, 2006). Wang et al. (2022) also noted that identification with Chinese culture is shaped in part by living environments and broader social contexts. From this perspective, “sharing” may involve social experiences that are associated with feelings of belonging and connectedness, which can support self-definition and identification processes (Alden et al., 2010).

The results further indicated that feel experience was not significantly associated with cultural identity. One possible explanation is that cultural identity involves cognitive, group-based, and affective components. Emotional responses alone—especially if they are immediate and transient—may not be sufficient to align with deeper recognition of cultural connotations. This pattern suggests that identity-related outcomes may be more closely linked to experiences that involve meaning-making grounded in cultural cognition, rather than to affective arousal alone.

Sense and act experiences were also not significantly associated with cultural identity in our model, which differs from findings reported by Altiparmakogullari and Hasirci (2023) and Deng et al. (2023). This discrepancy may relate to differences in the cultural carrier and experience composition. In our study, the carrier was cultural and creative tourism products, where sensory interaction is often dominated by sight, sound, and touch. In contrast, prior work has included additional sensory channels (e.g., smell and taste), which may relate to richer affective engagement. Furthermore, in Deng et al., interactive experiences incorporated perceived value and cultural meanings more explicitly, whereas the products in our setting may have emphasized hands-on craftsmanship processes that are intuitive and accessible but may not always convey deeper cultural connotations, potentially weakening identity-related appraisal.

Taken together, these results suggest that cultural identity is more strongly linked to multidimensional experiences that combine meaning-oriented reflection and social connectedness, rather than to sensory or affective experiences in isolation. In this sense, think experience may correspond to deeper interpretation of cultural meanings, while relate experience may correspond to identity processes shaped by social belonging. By contrast, sense, act, and feel experiences may require additional design elements that make cultural connotations more salient in order to align more closely with cultural identity.

In practical terms, these findings highlight two design considerations for supporting cultural identity through CCTPD: (1) incorporating cultural connotations—especially value-based and symbolic resources—that invite deeper reflection; and (2) strengthening the social functions of cultural and creative tourism products to facilitate sharing and belonging. For example, cultural creative attractions may present traditional culture through approachable IP imagery, and designers may provide interactive platforms and user communities to encourage constructive cultural sharing.

#### 6.3.3 SENSE, RELATE, and FEEL experience design are positively associated with cultural confidence

Beyond cultural cognition, our results indicate that sense experience was also positively associated with cultural confidence. Illustrative qualitative evidence from on-site interactions and visitor feedback suggests that some visitors reported strong feelings of cultural pride in response to ceramic cultural creative products displayed at the research site. Compared with many other cultural and creative tourism products, these ceramic products were perceived as exquisitely crafted, and the exhibition design conveyed a salient cultural atmosphere. Such sensory cues may be linked to visitors’ appreciation of traditional Chinese culture and artistic aesthetics and may align with their expectations (Gao and Xu, 2022). In this sense, sensory engagement may co-occur with feelings of awe and emotional impact, which may relate to higher reported cultural confidence.

With regard to relate experience and cultural confidence, prior research suggests that individuals often use others’ evaluations as informational cues when assessing their own abilities (Dennis, 1999). From this perspective, when visitors share cultural experiences and receive positive feedback from others, they may perceive recognition of their cultural literacy and aesthetic taste. Such perceived recognition may function as a form of psychological support and may be associated with stronger cultural confidence.

Consistent with Zhou et al. (2022) and Lin et al. (2022), feel experience was also positively associated with cultural confidence, potentially because affective resonance and self-relevant cultural appraisal tend to align with pride and affirmation toward one’s culture. In our setting, the Three Gorges Museum is widely recognized as a national-level museum and as a salient symbol of Bashu cultural origins. This place-based symbolism may provide context that is conducive to positive cultural appraisal and may be linked to higher cultural confidence.

By contrast, act experience and think experience were not significantly associated with cultural confidence in this study. One possible explanation is that these dimensions are more strongly oriented toward rational or knowledge-focused engagement and may involve less immediate affective appraisal. This interpretation is also consistent with the mediation results, where cultural cognition alone did not show a significant direct association with cultural confidence.

In summary, at the stage most closely related to cultural confidence, the salience of sense, relate, and feel experiences suggests that affective and identity-relevant engagement may be particularly important. From a design perspective, cultural and creative tourism products might leverage audiovisual and interactive technologies to enrich sensory cues and strengthen immersion. It may also be beneficial to create channels for visitors to share experiences and receive positive social feedback, which may support identity affirmation. Moreover, selecting cultural carriers with deeper symbolic and value-based meanings may help elicit emotional resonance, which is often associated with cultural confidence.

### 6.4 Implications

This study enriches the theoretical foundations of cultural psychology and design research. First, cultural confidence is a concept that has been predominantly discussed in the Chinese context. To our knowledge, existing studies have rarely examined the proposed psychological formation of cultural confidence using empirical model testing, with much prior work focusing on dimensions rather than the relationships and processes linking cognition, identity, and confidence. In our model, the indirect-effect results highlight the central role of cultural identity in the cognition–identity–confidence framework. Specifically, the full mediation pattern suggests that cultural cognition was not significantly associated with cultural confidence when cultural identity was included, whereas cultural identity showed a strong association with cultural confidence. The sequential indirect effect further indicates that CCTPD is associated with cultural confidence partly through its associations with cultural cognition and cultural identity. Taken together, these patterns are consistent with the view that cultural communication may relate to cultural confidence through an interplay of cognitive meaning-making and identity-relevant (affective/value-based) appraisal, offering a refined framework for understanding how cultural dissemination can be linked to cultural confidence.

For a concrete illustration, participatory museum practices can help translate the proposed framework into visitors’ lived experiences. In museums such as the Chongqing China Three Gorges Museum, programs (e.g., calligraphy workshops) can invite visitors to practice brush techniques while learning the cultural meanings behind characters and scripts. Such activities may simultaneously support cultural cognition (acquiring historical and symbolic knowledge) and cultural identity (experiencing shared cultural practice and belonging), which in turn may relate to stronger cultural confidence. Similar initiatives—such as traditional craft demonstrations, guided storytelling tours, and interactive heritage displays—illustrate how CCTPD can translate abstract cultural narratives into embodied, place-based experiences that support identity processes.

These findings provide a more comprehensive explanatory lens for cultural psychology and broaden the perspective on cultural cognition by underscoring that cultural communication and public education may benefit from going beyond the accumulation of factual information to include identity-relevant and emotionally resonant elements. Accordingly, in policy-making and social practice, cultural communication may be more effective when it moves beyond information transmission toward value-based identification and shared meaning-making. Public cultural projects may therefore prioritize depth of engagement rather than only expanding reach. In cultural product design, designers may consider combining knowledge-oriented content with emotionally engaging features and social sharing opportunities so that cognition- and identity-related processes can jointly align with cultural confidence.

**Figure 6:**
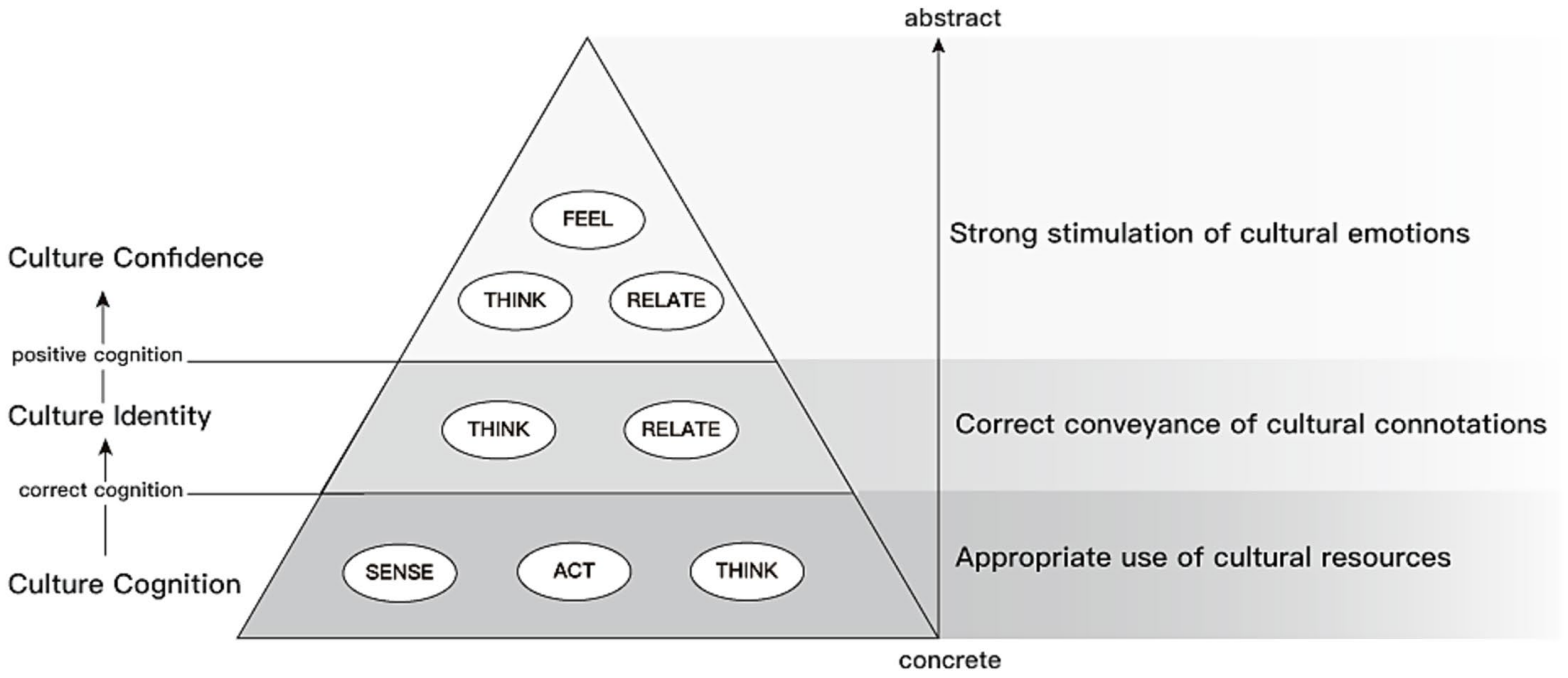
Synthesis: links between cultural-confidence mechanism (cognition → identity → confidence) and CCTPD dimensions (sense/act/think/relate/feel).

Second, this study addresses the gap in understanding how CCTPD relates to cultural confidence. Our results suggest that CCTPD, as a design medium in a museum context, is positively associated with cultural cognition, cultural identity, and cultural confidence through multiple direct and indirect pathways. We also examined how CCTPD’s experiential dimensions relate to different stages of the proposed psychological process. As illustrated in Figure 6, different experiential dimensions showed distinct associations with cognition, identity, and confidence in a layered manner. This pattern is broadly consistent with Maslow’s hierarchy-of-needs perspective, in the sense that visitors’ needs may range from more basic, information-oriented engagement to deeper identity- and value-related experiences. Specifically, the cultural cognition stage is oriented toward the acquisition and transmission of cultural knowledge and the activation of learning interest; the cultural identity stage emphasizes interpretation of cultural connotations and social/collective meanings; and cultural confidence appears to align with both cognitive and affective foundations.

Notably, the same experiential design dimension may correspond to different psychological stages. For example, sense experience may be more strongly linked to cultural cognition when cultural resources are made salient through design transformation, while an immersive cultural atmosphere may be more closely associated with cultural confidence. Think experience may relate to cultural cognition through educational, knowledge-oriented content, while imagination- and memory-evoking designs may be more closely associated with cultural identity. Relate experience may support cultural identity through social belonging, whereas social recognition of cultural literacy and aesthetic taste may align more with cultural confidence. These insights provide actionable directions for designers and policymakers and highlight the importance of considering different stages of the cognition–identity–confidence framework when developing experience strategies.

### 6.5 Limitations and directions for future research

This study has several limitations, which also suggest directions for future research.

First, the association between cultural cognition and cultural confidence may vary depending on the cultural carrier and the specific content through which culture is communicated. Future studies could test whether the proposed relationships hold across alternative carriers, such as traditional theatre, intangible cultural heritage crafts, and digital media. Such comparisons would help clarify the roles of different carriers in linking cultural dissemination to psychological outcomes and broaden perspectives in cultural communication research.

Second, this study examined cultural cognition, cultural identity, and cultural confidence as higher-level constructs to test their relationships within the proposed framework. Although the questionnaire items were developed based on relevant theories and were designed to reflect the multidimensional nature of these constructs, the present analyses did not further disentangle or compare specific subdimensions. Future research could therefore investigate the dimensional structures of cultural cognition, cultural identity, and cultural confidence in more detail and examine how different facets interact across stages, thereby enriching the theoretical account of cultural confidence formation.

Third, we did not explicitly examine whether the proposed relationships differ across demographic groups. Future studies could explore how demographic factors (e.g., age, cultural background, and education level) relate to each stage of cultural confidence formation and whether these factors moderate the links among cultural cognition, cultural identity, and cultural confidence. In addition, investigating users’ heterogeneous preferences for CCTPD may support more targeted and inclusive design strategies for different visitor segments and contribute to a more comprehensive design methodology.

Finally, because the sample was drawn exclusively from visitors to a single site (the Cultural and Creative Experience Pavilion of the Chongqing Three Gorges Museum), the external validity of the findings may be constrained. Future research should replicate the model using multi-site samples (e.g., different museum types and regions) and cross-cultural datasets to examine whether the cognition–identity–confidence pattern generalizes across cultural and institutional contexts. Moreover, due to the open nature of the research site and limitations in manpower and resources, convenience sampling was used. Although procedural measures were implemented to reduce potential biases (e.g., pretest-based item refinement, standardized instructions, trained survey administrators, and sampling across different time periods), the sample may still be subject to selection bias and may not fully represent the overall museum visitor population (e.g., the proportion of female and 18–24-year-old respondents was noticeably higher than that of other demographic groups).

In addition, some items exhibited a tendency toward high-end responses, which may reflect ceiling effects and/or social desirability. Although key indirect effects were estimated using bootstrapping and appeared robust, future work could improve measurement sensitivity by incorporating reverse-coded items and triangulating self-reports with behavioral indicators (e.g., observed engagement, revisit intention, and sharing behavior). Nevertheless, the study primarily aims to test the proposed relationships; thus, the observed patterns provide preliminary evidence, and the magnitude and generalizability of effects should be interpreted with caution. Because the data are cross-sectional, the sequential mediation should be interpreted as correlational rather than causal; longitudinal or experimental designs are needed to test temporal precedence and causal pathways. Future studies could also consider more rigorous sampling strategies (e.g., random or stratified sampling), alternative recruitment channels (e.g., social media combined with on-site recruitment), larger samples, and moderation analyses to strengthen robustness and generalizability.

## 7 Conclusion

Taken together, the findings provide preliminary support for the following conclusions.

Amid urban regeneration and intensifying tourism flows, place-based creative-product experiences in museums may be associated with stronger cultural identity, which in turn may relate to higher cultural confidence and pro-museum intentions. The findings are consistent with a sequential pattern in which cultural cognition is positively associated with cultural identity, which is then positively associated with cultural confidence. Although cultural cognition provides a cognitive basis for acquiring cultural knowledge, it showed no significant direct association with cultural confidence in the tested model. Cultural identity appears to function as a key mediator, linking cognitive understanding with affective resonance and pride, which are associated with higher cultural confidence.

Cultural and creative tourism product design (CCTPD) was positively associated with cultural confidence through multiple direct and indirect pathways, with cultural identity playing a central mediating role across pathways. These results suggest that, in designing cultural and creative tourism products, combining knowledge-oriented elements with identity-relevant and emotionally resonant experiences may be beneficial for supporting cultural confidence.

Different experiential dimensions of CCTPD showed distinct associations with cultural cognition, cultural identity, and cultural confidence. As experiential attributes extend from sensory engagement to more reflective and relational experiences, the corresponding psychological stages linked to cultural confidence also tend to deepen. This underscores the value of multidimensional and targeted experience design strategies.

## Data Availability

The original contributions presented in the study are included in the article/Supplementary material, further inquiries can be directed to the corresponding authors.
